# Frequency Specific Cortical Dynamics During Motor Imagery Are Influenced by Prior Physical Activity

**DOI:** 10.3389/fpsyg.2018.01976

**Published:** 2018-10-25

**Authors:** Selina C. Wriessnegger, Clemens Brunner, Gernot R. Müller-Putz

**Affiliations:** ^1^Institute of Neural Engineering, Graz University of Technology, Graz, Austria; ^2^BioTechMed-Graz, Graz, Austria; ^3^Institute of Psychology, University of Graz, Graz, Austria

**Keywords:** motor imagery (MI), EEG, ERD/ERS, motor execution (ME), training effects

## Abstract

Motor imagery is often used inducing changes in electroencephalographic (EEG) signals for imagery-based brain-computer interfacing (BCI). A BCI is a device translating brain signals into control signals providing severely motor-impaired persons with an additional, non-muscular channel for communication and control. In the last years, there is increasing interest using BCIs also for healthy people in terms of enhancement or gaming. Most studies focusing on improving signal processing feature extraction and classification methods, but the performance of a BCI can also be improved by optimizing the user’s control strategies, e.g., using more vivid and engaging mental tasks for control. We used multichannel EEG to investigate neural correlates of a sports imagery task (playing tennis) compared to a simple motor imagery task (squeezing a ball). To enhance the vividness of both tasks participants performed a short physical exercise between two imagery sessions. EEG was recorded from 60 closely spaced electrodes placed over frontal, central, and parietal areas of 30 healthy volunteers divided in two groups. Whereas Group 1 (EG) performed a physical exercise between the two imagery sessions, Group 2 (CG) watched a landscape movie without physical activity. Spatiotemporal event-related desynchronization (ERD) and event-related synchronization (ERS) patterns during motor imagery (MI) tasks were evaluated. The results of the EG showed significant stronger ERD patterns in the alpha frequency band (8–13 Hz) during MI of tennis after training. Our results are in evidence with previous findings that MI in combination with motor execution has beneficial effects. We conclude that sports MI combined with an interactive game environment could be a future promising task in motor learning and rehabilitation improving motor functions in late therapy processes or support neuroplasticity.

## Introduction

Motor Imagery (MI) is one task which has been used for driving brain plasticity, skill acquisition, and motor learning in several fields including sports, brain-computer interface (BCI) research, and motor rehabilitation (Jackson et al., 2001; Braun et al., 2006; Vries and Mulder, 2007; Guillot et al., 2008; Munzert and Zentgraf, 2009; Neuper et al., 2009; Sharma et al., 2009; Dijkerman et al., 2010; Ietswaart et al., 2011; Lee et al., 2011; Schuster et al., 2011; Silvoni et al., 2011; Kaiser et al., 2012; Malouin et al., 2013). MI describes the mental simulation of voluntary movement without its actual execution (Jeannerod, 1995). In the past years, MI has become a well-established complementary method for motor skill learning (Pascual-Leone et al., 1995; Gentili et al., 2006, 2010; Munzert et al., 2009; Schuster et al., 2011; Ruffino et al., 2017). Additionally, MI training has similar effects like motor training. Both result in a more focused, specific activation of the underlying cortical areas and an increase in motor performance after training sessions (Nyberg et al., 2006; Gentili et al., 2010; Reiser et al., 2011; Zhang et al., 2011; Kaiser et al., 2014). This is in line with the core message of the “simulation hypothesis” by Jeannerod (1994), claiming that the mental rehearsal of a movement activates the same cortical areas as actual execution. The rehearsal of motor actions through physical and mental practice can induce brain changes (plasticity) associated with skill learning and has already been demonstrated in animal models (e.g., Nudo et al., 1996) and in humans (Pascual-Leone et al., 1995; Lafleur et al., 2002; Jackson et al., 2003). For example Pascual-Leone et al. (1995), observed that changes in cortical patterns over sensorimotor areas after mental training are similar to those obtained with physical training. Furthermore, several studies suggested that MI facilitates skill acquisition and motor learning in a manner similar to physical practice resulting in plastic changes in the brain following repetitive mental practice (Grezes and Decety, 2001; Miller et al., 2010; Sharma and Baron, 2013). Therefore MI is a commonly used strategy for improving motor learning in a variety of sports (Brouziyne and Molinaro, 2005; Owen et al., 2006; Olsson et al., 2008; Guillot et al., 2013). In sports athletes of different disciplines commonly use MI to strengthen kinesthetic memory between training sessions maintaining their performance level or to stabilize complex routines (Rodgers et al., 1991; Murphy, 1994; Rushall and Lippman, 1998). Very often they imagine their forthcoming performance in real time routines to “get a feeling” for how to respond to the requirements of a certain task (Munzert and Lorey, 2013). For example Olsson et al. (2008) investigated the role of task familiarity and task complexity in a group of high jumpers and novices. They found that the activation of motor regions related to the task strongly depends on a well-established motor representation resulting from physical training.

Previous work on MI has already pointed out the significant influence of the task used on the neural response: which body parts need to be imagined (Parsons, 2001; Ehrsson et al., 2003; Szameitat et al., 2007a) or which MI strategy, kinaesthetic vs. visual or first-perspective vs. third-perspective (Neuper et al., 2005; Fourkas et al., 2006; Guillot et al., 2009; Stecklow et al., 2010; Hétu et al., 2013). Previous MI studies primarily used simple finger, hand and foot movements (Gerardin et al., 2000; Lotze and Halsband, 2006; Erbil and Ungan, 2007; Wriessnegger et al., 2008) or finger to thumb opposition tasks (Porro et al., 1996; Solodkin et al., 2004) only a few deal with more complex tasks like sports MI or tool use (Owen et al., 2006; Bakker et al., 2007; Olsson et al., 2008; Guillot et al., 2013; Kalicinski and Raab, 2013; Wriessnegger et al., 2014, 2016). For example a study by Szameitat et al. (2007b) showed that using more vivid and familiar tasks not only drives comparable cortical networks to those for a simple task, but also that they are more effective. They investigated MI of complex daily movements like brushing the hair, dancing in a club, playing cards or buttoning a shirt from a kinesthetic first-person perspective. According to their findings it is assumed that MI is effective since it activates a similar cortical network to that of physical training, but more importantly they pointed out the advantage of the task itself. For example using activities of the daily life for the imagination is simply for every participant since everybody is familiar with these tasks and is able to generate vivid, first-person imagery without prior training. Beside the important application of MI in motor skill learning and motor rehabilitation MI is often used inducing changes in electroencephalographic (EEG) signals for imagery-based brain-computer interfacing (BCI). A BCI is a device translating brain signals into control signals providing severely motor-impaired persons with an additional, non-muscular channel for communication and control. Over the past 25 years many studies have shown that brain activity changes associated with motor imagery can serve as useful control signals for BCIs (Pfurtscheller and Neuper, 2001; Pfurtscheller et al., 2003; Allison et al., 2010; McFarland and Wolpaw, 2011; Wolpaw and Wolpaw, 2012; Friedrich et al., 2013; Alonso-Valerdi et al., 2015; Vasilyev et al., 2017). Importantly, in the last years BCI research have become more and more interesting for a broader community of researchers, due to the shift from an application for disabled to healthy users (Allison et al., 2007; Blankertz et al., 2012). There are different ways to improve the performance of BCIs but most studies focused on signal processing, classification, and feature extraction (Alimardani et al., 2017; Kevric and Subasi, 2017; Wankar et al., 2017) However, BCI performance can also be improved by changing common control strategies of the user. That is, using more intuitive mental tasks for control (Obermaier et al., 2001; Curran and Stokes, 2003; Lotte et al., 2013; Lotte and Cichocki, 2017; Ma et al., 2017; Qiu et al., 2017; Jeunet et al., 2018). Focusing on motor imagery as the proper mental strategy we (Wriessnegger et al., 2014) reported an fMRI study where participants imagined playing tennis or soccer, after a short training session in both disciplines. We showed that only 10 min of training are sufficient to boost motor imagery patterns in related brain regions including the premotor cortex, supplementary motor area (SMA), fronto-parietal regions, and subcortical structures. Furthermore, all participants reported that it was easier to imagine the requested type of sport after the exercise, because of its vividness in memory. The results of this study motivated us to partly replicate it by means of EEG. The ultimate motive by using more vividt asks in future motor imagery-based BCI systems is to improve MI pattern classification or support motor rehabilitation in final stages.

In the present study participants were divided in two groups, one experimental group (EG), performing a motor exercise between the two imagery sessions (PRE: before physical training; POST: after physical training); one control group (CG), watching a movie between the two motor imagery sessions, without any physical intervention. Both groups performed two MI tasks with varying vividness of imagery, namely squeezing a ball and playing tennis.

Based on our previous work we expected stronger ERD/S patterns for the EG in the POST condition compared to the PRE condition due to the physical intervention. That is a more pronounced cortical activity will be elicited due to the intensification of the imagery task after physical exercise. Additionally, we hypothesize that both groups show a more distributed stronger brain activity during MI of playing tennis, due to its more complex and engaging task.

## Materials and Methods

### Participants

Thirty healthy right-handed students gave informed consent to participate in the study. All reported normal or corrected to normal vision and none of them had a history of psychiatric or neurological disorders. Participants were matched with regard to sex and age, and they were randomly assigned to the control group (*n* = 15; mean age: 24.9; range: 20–30 years; 7 women and 8 men) or to the experimental group (*n* = 15; mean age: 24.8; range: 20–28 years; 7 women and 8 men). 70 % of the participants regularly perform different kinds of sports and only five of them play tennis. The study was approved by the local ethics committee (Medical University of Graz) and is in accordance with the ethical standards of the Declaration of Helsinki. After a detailed written and oral instruction participants gave informed written consent to participate in the study. They received financial compensation for their participation.

### Experimental Procedure

Participants were seated in a comfortable armchair in a soundproof, air-conditioned, and dimmed cabin. The distance between the participants and the monitor presenting the experimental paradigm was approximately 120 cm. A webcam was positioned above the monitor to observe participants’ behavior from outside the cabin during the experiment.

**FIGURE 1:**
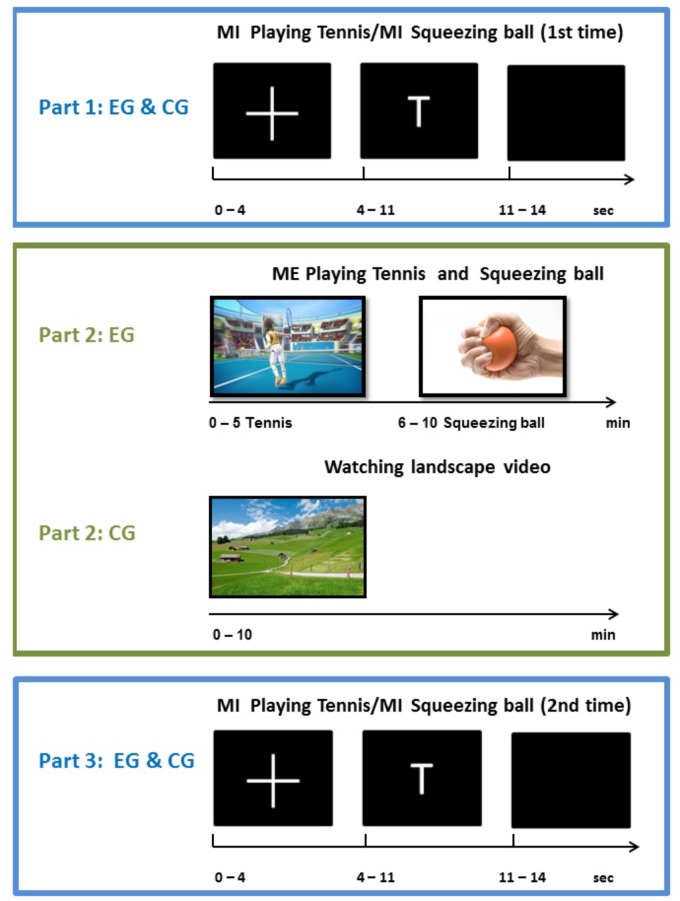
Schematic illustration of experimental design and timing of one trial.

The experimental procedure encompassed a pre-measurement, the execution or relaxing intervention, and a post-measurement. During the pre-measurement, participants from both groups performed the motor imagery task according to the written instructions. Whenever the letter “T” appeared on the screen in front of them, participants had to imagine playing tennis for 6 s repetitively. More concretely, they had to imagine repeatedly returning balls with their right fore hand from a first person perspective. In case of the displayed letter “H,” they had to imagine repeatedly squeezing a ball for 6 s with their right hand. Participants had to perform the corresponding task as long as the letter was on the screen. Both groups were instructed to perform a kinesthetic imagery of the task, characterized by ‘feeling’ the movements and muscles activations generating kinesthetic impressions. Each type of imagery was performed 15 times per run in a pseudo-randomized order. In total four runs were performed, resulting in 120 trials (60 trials squeezing ball imagery, 60 trials playing tennis imagery). During the intervention phase, participants from the experimental group played tennis via “Kinect” and squeezed a ball for 5 min each. These execution interventions were also performed in randomized order within the experimental group. Participants assigned to the control group watched a movie about landscapes for 10 min and had no physical exercise. After this intervention phase, participants of both groups performed the two imagery tasks again (playing tennis or squeezing a ball) inside the electrically shielded cabin. The whole experimental procedure and the exact timing of one trial are shown in Figure 1.

One trial lasted about 14 s, consisting of a fixation cross (4 s), the imagery phase (6 s) and a rest period (4 s). One run consisted of 30 trials (15 per imagery task) with four runs in the pre-measurement phase (Part 1) and 4 runs in the post-measurement phase (Part 3). In total each participant performed eight runs with 240 trials.

### EEG Recording

**FIGURE 2:**
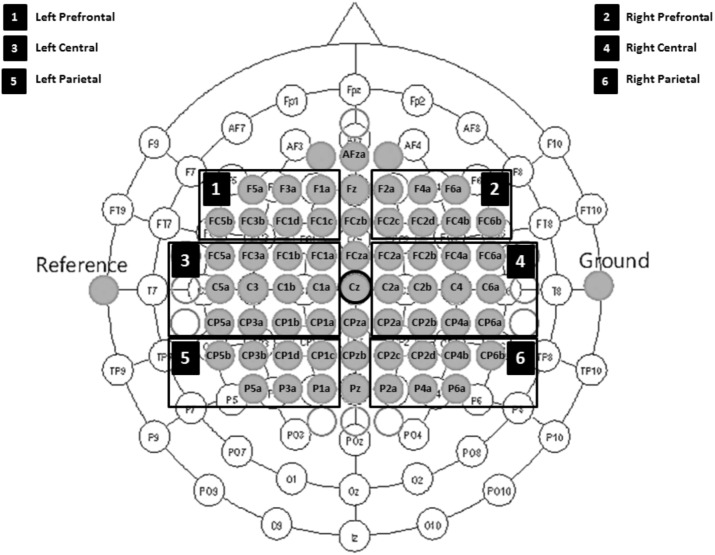
Electrode montage setup including six defined ROIs. 1, left prefrontal; 2, right prefrontal; 3, left central; 4, right central; 5, left parietal; and 6, right parietal regions.

Electroencephalographic was recorded with Ag/AgCl sintered ring electrodes mounted in an elastic cap (Easy Cap) from 60 scalp locations according the 10/10 international system with an extended customized montage setup (see Figure 2).

EOG was recorded with three electrodes (two placed at the outer canthus of each eye and one at the glabella). The reference and ground electrodes were placed on the left and right mastoid, respectively. All signals were recorded using two BrainAmp amplifiers (Brainproducts, Gilching, Germany) with a sampling rate of 1000 Hz and a band-pass filter between 0.1 and 100 Hz. The BrainVision Recorder application (Brainproducts, Gilching, Germany) stored the raw biosignal data together with the digital trigger signals from the stimulus presentation program, which was implemented in MATLAB (Mathworks, Natick, United States).

### EEG Preprocessing and ERD/ERS Analysis

First, we down sampled the raw EEG data to 250 Hz and re-referenced all channels to Cz. We manually inspected the continuous EEG signals and marked segments containing artifacts, which we discarded in all subsequent analyses. Next, we used non-causal FIR bandpass filters to extract time signals in the bands 8–13 Hz (alpha band) and 16–24 Hz (beta band). Squaring the samples resulted in continuous bandpower time series, which we then used to create epochs around the cues for hand and tennis imagery. We considered segments from -3.5 to 3.5 s relative to each cue for our ERD/ERS calculation, where the baseline and activation intervals ranged from -3.5 to -0.5 s and 0.5 to 3.5 s, respectively. Finally, we computed ERD/ERS values for each group (experimental and control), condition (hand and tennis imagery), frequency band (alpha and beta), time point (pre- and post-measurements), and channel separately according to the equation ERDS = ((*A*%*R*) – 1) 100%, where *A* is the average band power in the activity time interval and *R* is the average band power in the baseline (or reference) time interval, both values averaged across epochs. Finally, we averaged groups of channels into the following six regions of interest: prefrontal left (F5a, F3a, F1a, FC5b, FC3b, FC1d, and FC1c), prefrontal right (F2a, F4a, F6a, FC2c, FC2d, FC4b, and FC6b), central left (FC5a, FC3a, FC1b, FC1a, C5a, C3, C1b, C1a, CP5a, CP3a, CP1b, and CP1a), central right (FC2a, FC2b, FC4a, FC6a, C2a, C2b, C4, C6a, CP2a, CP2b, CP4a, and CP6a), parietal left (CP5b, CP3b, CP1d, CP1c, P5a, P3a, and P1a), and parietal right (CP2c, CP2d, CP4b, CP6b, P2a, P4a, and P6a) (see Figure 2).

We computed time/frequency ERD/S maps similar to the procedure used to calculate ERD/S values. Specifically, we used an FFT-based sliding window approach with 513 frequency bins and 128 time points within a time range of -4 to 7 s relative to the cue. Significance of time/frequency values was determined via a bootstrap approach.

### Statistical Analysis

In order to investigate the potential influence of the task and the timing on the ERD/S patterns, we performed several 2 × 2 × 6 repeated measures analyses of variance (ANOVA) using IBM SPSS Statistics 23. ERD/S values were analyzed for each group (EG, CG) and both frequency bands (alpha and beta) separately considering the variables “TASK” (2 levels: MI Tennis and MI Ball squeezing), “TIME” (2 levels: PRE and POST), and ROI (6 levels: prefrontal left, prefrontal right, central left, central right, parietal left, and parietal right) as within-subject variables. A Greenhouse-Geisser correction was applied whenever Mauchly’s test indicated a lack of sphericity. For *post hoc* analysis, we used FDR-corrected *p*-values at *p* < 0.05 to control for multiple comparisons (Benjamini and Hochberg, 1995). We also verified that baseline band power values between the two groups were not significantly different to ensure that ERD/S differences are not confounded by baseline differences. In the following section, we report the results of each group separately.

## Results

Before running the statistical analysis, we analyzed the baseline band power values. Overall, we found no differences between the baseline band power values of the two groups (EG, CG). In particular, we computed mixed ANOVAs with the within-factor “ROI” and the between-factor “GROUP” separately for each of the two frequency bands, time points, and tasks. As expected, we found baseline differences across ROIs, but not across the two groups. This reliability check of the data enabled us to continue with the ERD/S analysis (details in Supplementary Material). The remaining results are organized as follows. Sections “Results EG Alpha Band” and “Results EG Beta Band” describe the ERD/S results of the EG for the alpha and beta bands, whereas sections “Results CG Alpha Band” and section “Results CG Beta Band” present the ERD/S results of the CG for the alpha and beta bands, respectively. Finally, a visualization of time-frequency maps of two exemplarily participants are shown in section “Exemplary Time-Frequency Maps.”

### Results EG Alpha Band

**FIGURE 3:**
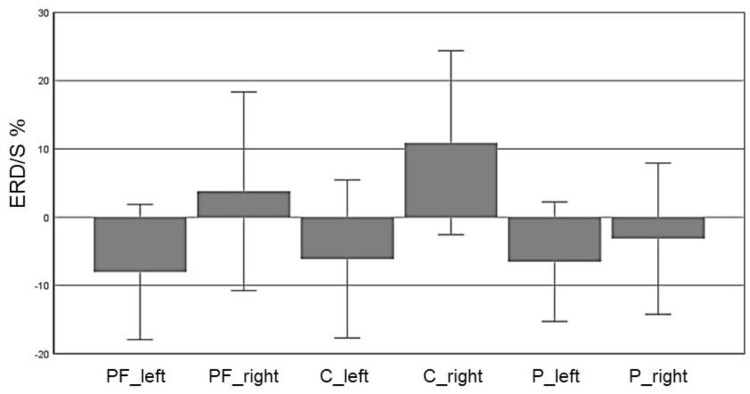
Profile Plot main effect ROI for the alpha band (error bars: ±2 SE).

In the alpha band (8–13 Hz), we observed a significant main effect for “ROI” (*F*(5,70) = 5.72, *p* = 0.007, η^2^ = 0.29), indicating a significant difference between the six ROIs: Prefrontal_left_ (*M* = -8.032, *SD* = 4.95), Prefrontal_right_ (*M* = 3.799, *SD* = 7.27), Central_left_ (*M* = -6.12, *SD* = 5.79), Central_right_ (*M* = 10.91, *SD* = 6.74), Parietal_left_ (*M* = -6.53, *SD* = 4.37) and Parietal_right_ (*M* = -3.15, *SD* = 5.54). Figure 3 shows the profile plot of the main effect ROI in the alpha-band for the EG.

**FIGURE 4:**
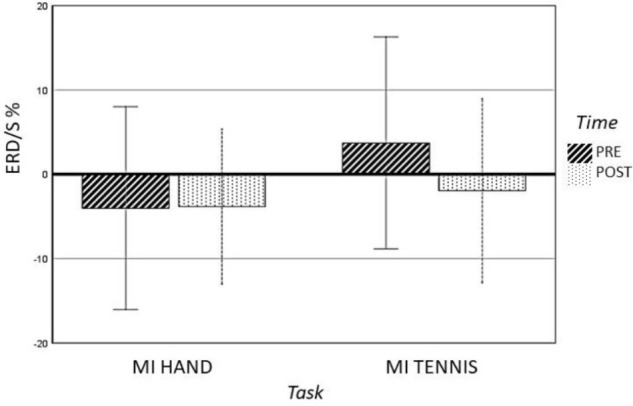
Profile plot of TASK × TIME interaction effect for the alpha-band (error bars: ±SE).

Furthermore, a significant interaction for TASK × TIME (*F*(1,14) = 4.85, *p* = 0.045, η^2^ = 0.25) was found (see Figure 4). The results of the paired-samples post-test showed a stronger ERD in the POST condition (after training) compared to the PRE condition only for tennis MI (*t*(14) = -2.378, *p* = 0.032). The MI of squeezing a ball (*M* = -3.84, *SD* = 4.59) revealed stronger ERD values compared to MI of tennis (*M* = -1.94, = 5.45) only in the PRE condition (*t*(14) = 3.265, *p* = 0.005). Figure 6 shows the topographical plot of mean ERD/S values for the E-Group in the alpha band (8–13 Hz).

**FIGURE 5:**
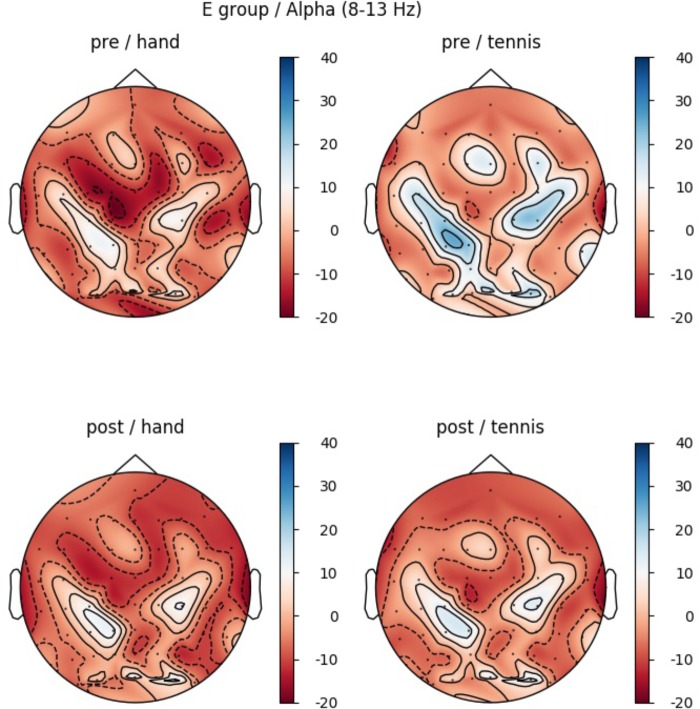
Topographical plot of mean ERD/S values for the E-Group in the alpha band (8–13 Hz). Red colors show ERD and blue colors ERS values. **Top:** ERDS activity before physical exercise (PRE-condition) for the hand and tennis task, respectively. **Bottom:** ERD/S activity after physical exercise (POST-condition) for the hand and tennis task, respectively.

*Post hoc* analysis showed significant differences between conditions Hand PRE and Tennis PRE over the following ROIs: central left (*t*(14) = -2.89, *p* = 0.012), central right (*t*(14) = -3.33, *p* = 0.005), parietal left (*t*(14) = -3.43, *p* = 0.004), and parietal right (*t*(14) = -3.09, *p* = 0.008). The results indicate stronger ERD in the PRE condition for the tennis MI task over all ROIs. Furthermore, a significant difference between condition Tennis PRE and Tennis POST was found over ROI central left (*t*(14) = 2.37, *p* = 0.032), indicating an increased ERD after training.

### Results EG Beta Band

**FIGURE 6:**
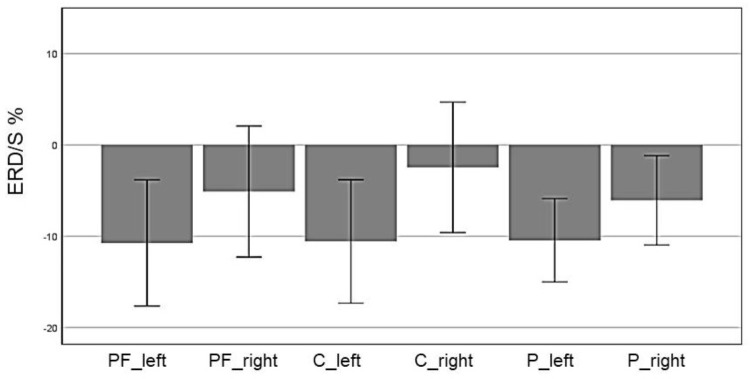
Profile Plot main effect ROI for the beta band (error bars: ±2 SE).

**FIGURE 7:**
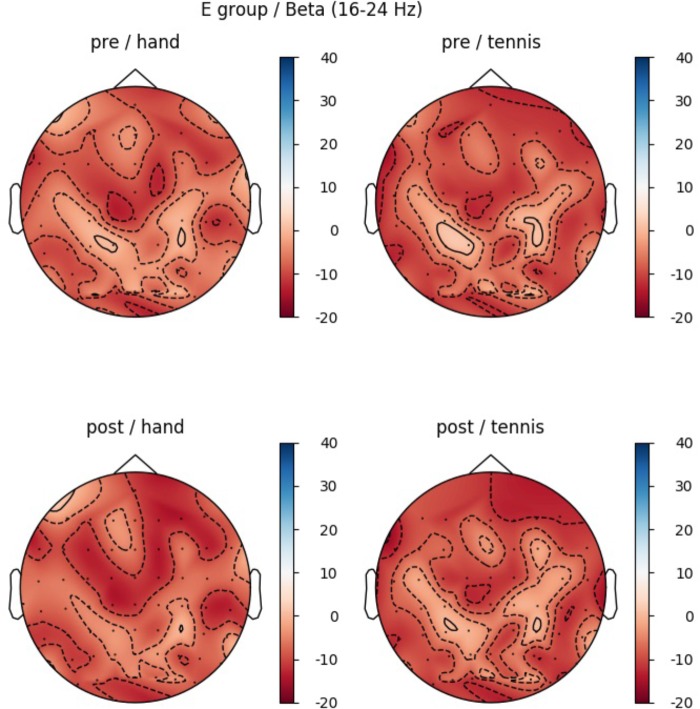
Topographical plot of mean ERD/S values for the E-Group in the beta band (16–24 Hz). **Top:** ERD/S activity before physical exercise (PRE-condition) for the hand and tennis task, respectively. **Bottom:** ERDS activity after watching the movie (POST-condition) for the hand and tennis task, respectively. Red colors indicate ERD and blue colors ERS values.

In the beta band (16–24 Hz), a significant main effect of ROI was found (*F*(5,70) = 5.85, *p* = 0.006, η^2^ = 0.28) indicating different ERD band power in the six defined ROIs: Prefrontal_left_ (*M* = -10.74, *SD* = 3.44), Prefrontal_right_ (*M* = -5.11, *SD* = 3.59), Central_left_ (*M* = -10.58, *SD* = 3.38), Central_right_ (*M* = -2.46, *SD* = 3.74), Parietal_left_ (*M* = -10.45, *SD* = 2.28), and Parietal_right_ (*M* = -6.08, *SD* = 2.45). Figure 6 shows the mean ERD/S% values for each ROI indicating a strong lateralization effect. Furthermore in Figure 7 the topographical maps of mean ERD/S values for the E-Group in the beta band (16–24 Hz) are shown.

### Results CG Alpha Band

**FIGURE 8:**
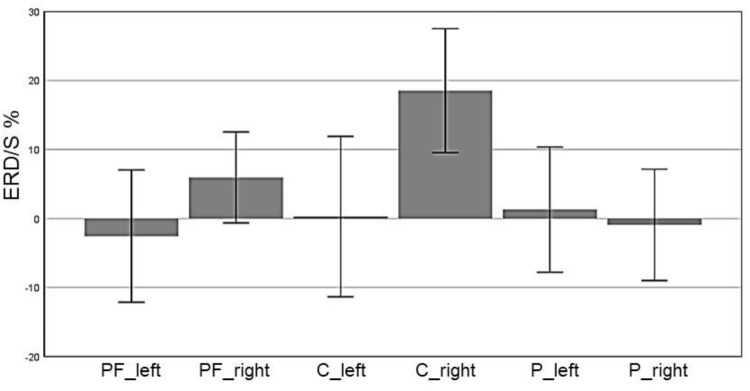
Profile Plot main effect ROI for the alpha-band (error bars: ±2 SE).

**FIGURE 9:**
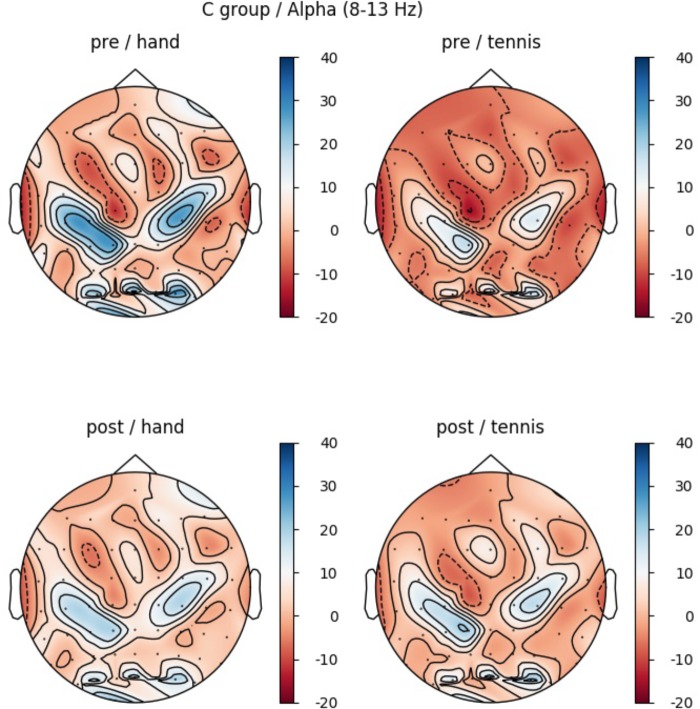
Topographical plot of mean ERD/S values for the C-Group in the alpha band (8–13 Hz). **Top:** ERDS activity before physical exercise (PRE-condition) for the hand and tennis task, respectively. **Bottom:** ERD/S activity after watching the movie (POST-condition) for the hand and tennis task, respectively. Red colors indicate ERD and blue colors ERS values.

In the CG, we observed a significant main effect in the alpha band for “ROI” (*F*(5,70) = 9.1, *p* ≤ 0.001, η^2^ = 0.4): Prefrontal_left_ (*M* = -2.55, *SD* = 4.79), Prefrontal_right_ (*M* = 5.95, *SD* = 3.27), Central_left_ (*M* = 0.28, *SD* = 5.83), Central_right_ (*M* = 18.52, *SD* = 4.49), Parietal_left_ (*M* = 1.31, *SD* = 4.53) and Parietal_right_ (*M* = -0.92, *SD* = 4.03). Figure 8 shows the profile plot of the main effect ROI in the alpha-band for the CG. The topographical plot of mean ERD/S values for the C-Group in the alpha band (8–13 Hz) is illustrated in Figure 9.

### Results CG Beta Band

**FIGURE 10:**
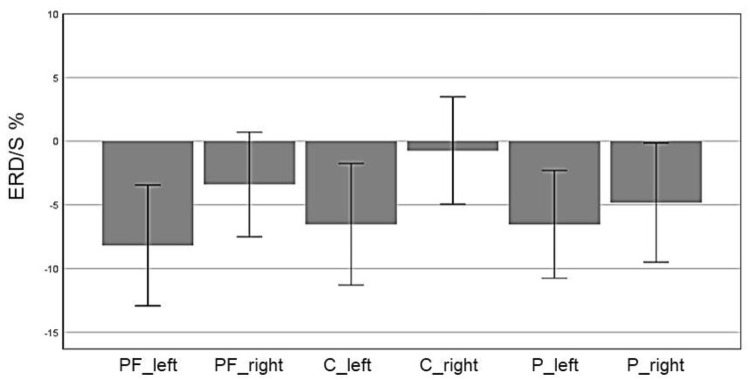
Profile Plot main effect ROI for the beta-band (error bars: ±2 SE).

**FIGURE 11:**
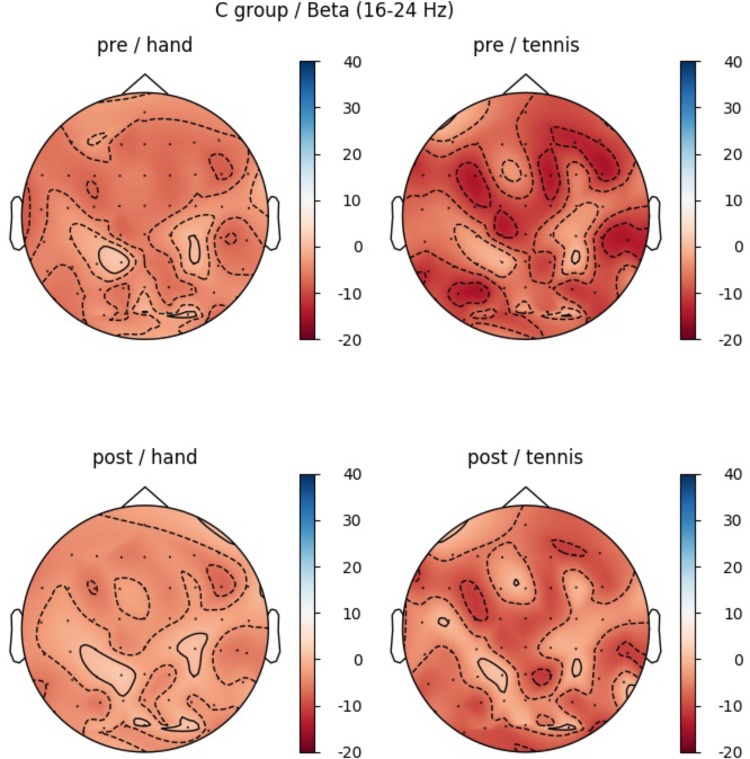
Topographical plot of mean ERD/S values for the C-Group in the beta band (16 – 24 Hz). **Top:** ERD/S activity in the first MI session (PRE-condition) for the hand and tennis task, respectively. **Bottom:** ERD/S activity of the 2nd MI session, after watching the movie (POST-condition), for the hand and tennis task, respectively. Red colors indicate ERD and blue colors ERS values.

In the beta band, the factor ROI (*F*(5,70) = 5.85, *p* = 0.004, η^2^ = 0.29) also reached significance: Prefrontal_left_ (*M* = -8.19, *SD* = 2.36), Prefrontal_right_ (*M* = -3.40, *SD* = 2.05), Central_left_ (*M* = -6.53, *SD* = 2.83), Central_right_ (*M* = -0.74, *SD* = 2.11), Parietal_left_ (*M* = -6.53, *SD* = 2.11), and Parietal_right_ (*M* = -4.81, *SD* = 2.34). In Figure 10 the profile plot of the main effect ROI for the beta-band is illustrated. The topographical plot of mean ERD/S values for the C-Group in the beta band (16–24 Hz)are illustrated in Figure 11.

Results indicate significant differences between left and right hemispheres for prefrontal and central regions due to the nature of the task. A comparison of the two group means, Control vs. Experimental group, revealed only significant results for Condition Hand PRE of the alpha band over ROI parietal right (*t*(14) = -2.17, *p* = 0.047).

### Exemplary Time-Frequency Maps

**FIGURE 12:**
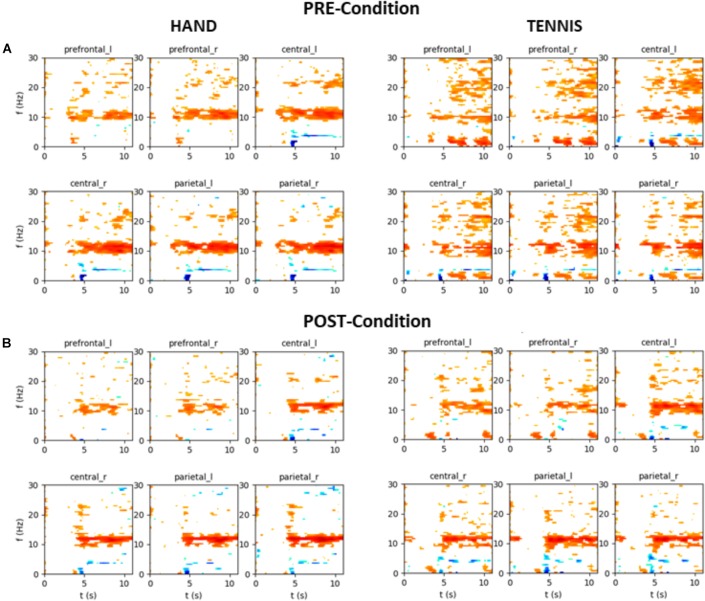
Mean time-frequency visualization of ERD/S for Subject S002 (EG group) over six ROIs, using the time interval between –3.5 and –0.5 s as the baseline. **(A)** ERD/S patterns in the PRE condition for the tasks “MI squeezing a ball” left side and “MI playing tennis,” right side. **(B)** ERD/S patterns in the POST condition (after physical exercise) for the tasks “MI squeezing a ball” left side and “MI playing tennis,” right side.

**FIGURE 13:**
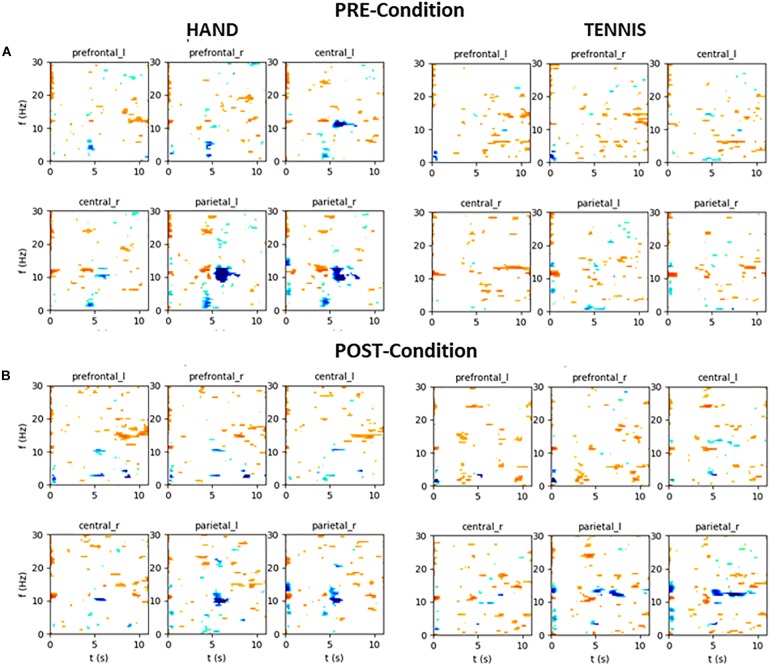
Mean time-frequency visualization of ERD/S for Subject S031 (the CG group) over six ROIs, using the time interval between –3.5 and –0.5 s as the baseline. **(A)** ERD/S patterns in the PRE condition for the tasks “MI squeezing a ball” left side and “MI playing tennis,” right side. **(B)** ERD/S patterns in the POST condition (after physical exercise) for the tasks “MI squeezing a ball” left side and “MI playing tennis,” right side.

In the following, the results of time-frequency maps of two exemplary subjects are illustrated. Figure 12 shows the results of participant S002 (EG), male, 28 years old and frequent soccer player. Figure 13 shows the results of participant S031 (CG), female, 23 years old and performs no sports at all. Both figures show the mean time-frequency visualization of ERD/S for the related subjects. In the top row (A) the ERD/S patterns in the PRE condition for the tasks “MI squeezing a ball” left side and “MI playing tennis,” right side are shown. In the bottom row (B) the ERD/S patterns in the POST condition (after physical exercise) for the tasks “MI squeezing a ball” left side and “MI playing tennis,” right side, are illustrated. Both have been randomly assigned to the different groups.

The alpha-band activity during MI of both tasks, “MI squeezing the ball” and “MI playing tennis” is stronger in the PRE condition for participant S002. It seems that after the training, the brain activity of the participant is more focused and efficient, resulting in a narrower banded activation. On the contrary, participant S031 shows a stronger alpha ERS pattern in the PRE condition “Hand” compared to the POST condition. For “Tennis” the alpha ERS pattern was stronger in the POST compared to the PRE condition. Overall the different ERD/S patterns of the two exemplary participants reflect the large individual differences in both tasks.

## Discussion

The present study investigated whether differences of brain activity during two MI tasks (squeezing a ball and playing tennis) evolve and specifically addressed the question if physical exercise of these tasks increases brain activation, concretely ERD/S patterns. Our results indicated different ERD/S activity in the alpha band for imaging of playing tennis between the two imaging sessions (PRE and POST) only in EG. The ERD was also stronger during MI of squeezing a ball, whereas the imagery of playing tennis elicited stronger ERS patterns in the PRE condition. As a main effect, we found significant differences between the six ROIs in both groups, EG and CG. A significant interaction of TIME × TASK was only observed in the EG. The results showed stronger ERD during MI of tennis in the POST compared to the PRE condition. This is in line with the results of the fMRI study by Wriessnegger et al. (2014), who found higher BOLD response in the primary motor cortex during MI after the physical exercise of playing tennis. Whereas they reported bilateral primary motor cortex activation after training, we found a clear contralateral ERD pattern in the left primary motor area.

It is well known that individuals differ in their ability performing certain motor tasks, therefore the skill level of an individual at a given time is a very important factor (Milton et al., 2004, 2007). It seems that squeezing a ball is so familiar to the individual that it is possible for them to generate a vivid mental representation without prior practice (Szameitat et al., 2007b). Compared to this simple movement, the MI task of playing tennis seems to be more complex and skill level dependent. Whereas in the EG group brain activity during MI playing tennis was smaller in the PRE condition, a stronger ERD pattern over regions related to the primary motor cortex left was already found in the CG. Taking a closer look to the demographic data of the participants, we found that unfortunately all five persons who regularly play tennis (minimum once a week), were randomly assigned to the CG. This fact might explain the stronger grand average ERD already in the PRE condition of this task in the CG illustrated in Figure 9.

This reduced ERD pattern in the POST condition of the CG can be interpreted in terms of efficiency that is after the first MI session participants were no longer novice to the task. Although they were not novice to the skill of playing tennis, they were novice in performing MI before the first session (PRE-condition). Previous literature found a correlation between degree of experience and brain activity (Haufler et al., 2000; Cremades, 2002; Kelly and Garavan, 2005; Milton et al., 2007; Bar and DeSouza, 2016; Guo et al., 2017). Researchers suggested that brain activation during novice performance reflects an inefficient and unorganized network which is indicative of the additional effort required. This effect is also visible in Figure 12A, showing the time-frequency map of one exemplarily person in the PRE condition TENNIS. The interpretation of the data in terms of neural efficiency is valid for both groups during the MI of squeezing a ball, since overall brain activity is reduced in the POST condition. As already mentioned the stronger ERD in the PRE and the reduced activation in the POST condition of CG during MI of playing tennis might be due to the fact, that five participants in this group regularly play tennis. Although statistical analysis did not support this assumption a possible effect could not be neglected and should be considered in future studies. It seems that the differences in ERD in the CG might be a combination of the lack of physical practice and the fact that some participants are familiar with the task of playing tennis.

Furthermore, our data clearly show high inter individual differences in ERD/S patterns in all tasks and groups (Doppelmayr et al., 1998). For example, the time-frequency visualization of ERD/S patterns of two exemplarily participants show distinct different ERD/S patterns for the same tasks. Whereas participant S002 (Figure 12) shows a stronger ERD during MI of squeezing a ball in almost all ROIs in the PRE condition, this pattern is reversed during MI of playing tennis. In the PRE condition the brain activity was more diffuse and widespread and after the training phase the ERD pattern was stronger and more focused in the alpha-band. On the contrary, participant S023 (Figure 13) shows a more diffuse pattern in both conditions and timings, with a strong ERD in the theta band but an ERS in the alpha band for MI condition playing tennis. This examples show the strong inter-individual differences in the alpha band during MI of tennis and squeezing a ball.

Comparing the topographic maps for the alpha band of both groups (Figures 5, 7) we observed an additional interesting pattern. In every condition an ERD over primary motor areas was found with a concurrent ERS pattern over sensorimotor regions which could be interpreted in terms of the “focal ERD/surround ERS” theory (Suffczynski et al., 2001; Pfurtscheller, 2003, 2006). This means that a desynchronization of rhythmic activity in the alpha band does not occur in isolation but can be accompanied by synchronization in neighboring cortical areas. Gerloff et al. (1998) reported a similar antagonistic behavior involving desynchronization of central mu rhythm and synchronization of parieto-occipital alpha rhythms during finger movement. This effect should be further investigated in future studies by applying special methods like estimations of cortical activity and dipole analysis.

One limitation in interpreting ERD/S changes between PRE and POST conditions in the present study is related with the level of expertise. For example, Kraeutner et al. (2018) suggested in a recent study with sports experts that cortical activity during MI is modulated by experience. It is assumed that controlling a given motor task is quite different for experts compared to novices. Thus, it is obvious that the skill level of the person who performs MI should be determined before interpretations of the possible effects of MI can be made. All our participants were naïve to MI but some of them were skilled in playing tennis. Our data suggested that prior knowledge of the task, like playing tennis, influences MI performance. The different skill levels regarding playing tennis between the EG and the CG might have influenced our results in the MI task of playing tennis. Future studies of neural correlates of MI should consider prior experience when selecting the task to be performed and the assignment to different groups.

## Conclusion

When acquiring or strengthen different skills through physical training cortical activity changes (Grezes and Decety, 2001). Thus it is likely that novice to a certain skill recruit different brain patterns to a different amount compared to trained participants or even experts (Guo et al., 2017). The current study provides evidence that a short physical exercise enhances neural correlates of MI in novice participants. We found stronger ERD patterns in the alpha band during MI of playing tennis after physical exercise in the EG. For MI of squeezing the ball this effect was not observed. Here no differences in alpha-band ERD between the PRE and POST condition was found which could be due to the less attractive, overlearned nature of the task. Considering the topographic distribution of ERD patterns in the alpha band the CG shows a controversial result. During MI of playing tennis stronger ERD patterns occurred in the PRE phase compared to the POST phase. This might be due to the fact that the skill level for playing tennis in this group was higher, that is, participants were not novices to the task. Future work of brain activity during MI should consider prior experience of the participants when selecting the task to be performed or should define some exclusion criteria.

## Author Contributions

SW, CB, and GM-P contributed to the conception and design of the study. SW and CB performed signal processing and the statistical analysis. SW wrote the first draft of the manuscript. CB wrote sections of the manuscript. All authors contributed to manuscript revision, read and approved the submitted version.

## Conflict of Interest Statement

The authors declare that the research was conducted in the absence of any commercial or financial relationships that could be construed as a potential conflict of interest.

## Supplementary Material

DATA SHEET S1 Statistical analysis of baseline differences between ROIs.
