# Supplementary material for: Frequency Specific Cortical Dynamics During Motor Imagery Are Influenced by Prior Physical Activity
Source: Front Psychol. 2018 Oct 25;9:1976. doi: 10.3389/fpsyg.2018.01976 (PMC6209646; doi:10.3389/fpsyg.2018.01976)
Supplement: DATA SHEET S1 — Statistical analysis of baseline differences between ROIs. [file Data_Sheet_1.DOCX]

> bl %>%

+ filter(band == "(8, 13)", time == "pre", task == "hand") %>%

+ ezANOVA(dv=bl, wid=id, within=.(roi), between=.(group))

$ANOVA

Effect DFn DFd F p p<.05 ges

2 group 1 28 0.01128405 9.161601e-01 0.0003424922

3 roi 5 140 5.84936936 6.130792e-05 * 0.0303554815

4 group:roi 5 140 0.71836444 6.106749e-01 0.0038299565

$`Mauchly's Test for Sphericity`

Effect W p p<.05

3 roi 0.004505511 7.397553e-23 *

4 group:roi 0.004505511 7.397553e-23 *

$`Sphericity Corrections`

Effect GGe p[GG] p[GG]<.05 HFe p[HF] p[HF]<.05

3 roi 0.3873294 0.00542314 * 0.4156239 0.004387601 *

4 group:roi 0.3873294 0.48789620 0.4156239 0.496857334

> bl %>%

+ filter(band == "(8, 13)", time == "pre", task == "tennis") %>%

+ ezANOVA(dv=bl, wid=id, within=.(roi), between=.(group))

$ANOVA

Effect DFn DFd F p p<.05 ges

2 group 1 28 0.007350669 9.322864e-01 0.0002250815

3 roi 5 140 6.964073311 7.731221e-06 * 0.0342131397

4 group:roi 5 140 0.595035847 7.037841e-01 0.0030177195

$`Mauchly's Test for Sphericity`

Effect W p p<.05

3 roi 0.002787226 2.491505e-25 *

4 group:roi 0.002787226 2.491505e-25 *

$`Sphericity Corrections`

Effect GGe p[GG] p[GG]<.05 HFe p[HF] p[HF]<.05

3 roi 0.3903045 0.002181926 * 0.4191735 0.001660931 *

4 group:roi 0.3903045 0.551102982 0.4191735 0.562426513

> bl %>%

+ filter(band == "(8, 13)", time == "post", task == "hand") %>%

+ ezANOVA(dv=bl, wid=id, within=.(roi), between=.(group))

$ANOVA

Effect DFn DFd F p p<.05 ges

2 group 1 28 0.2677892 6.088831e-01 0.008265080

3 roi 5 140 8.8660517 2.471850e-07 * 0.039127896

4 group:roi 5 140 0.4047532 8.448821e-01 0.001855557

$`Mauchly's Test for Sphericity`

Effect W p p<.05

3 roi 0.003415315 2.785782e-24 *

4 group:roi 0.003415315 2.785782e-24 *

$`Sphericity Corrections`

Effect GGe p[GG] p[GG]<.05 HFe p[HF] p[HF]<.05

3 roi 0.3592078 0.0007607391 * 0.3822714 0.0005664318 *

4 group:roi 0.3592078 0.6473397984 0.3822714 0.6599207393

> bl %>%

+ filter(band == "(8, 13)", time == "post", task == "tennis") %>%

+ ezANOVA(dv=bl, wid=id, within=.(roi), between=.(group))

$ANOVA

Effect DFn DFd F p p<.05 ges

2 group 1 28 0.1593415 6.927919e-01 0.004980206

3 roi 5 140 8.6579062 3.581312e-07 * 0.035916321

4 group:roi 5 140 0.5686289 7.239225e-01 0.002440798

$`Mauchly's Test for Sphericity`

Effect W p p<.05

3 roi 0.004756106 1.401549e-22 *

4 group:roi 0.004756106 1.401549e-22 *

$`Sphericity Corrections`

Effect GGe p[GG] p[GG]<.05 HFe p[HF] p[HF]<.05

3 roi 0.3715959 0.0007530944 * 0.3969197 0.0005499409 *

4 group:roi 0.3715959 0.5573881409 0.3969197 0.5682595683

> bl %>%

+ filter(band == "(16, 24)", time == "pre", task == "hand") %>%

+ ezANOVA(dv=bl, wid=id, within=.(roi), between=.(group))

$ANOVA

Effect DFn DFd F p p<.05 ges

2 group 1 28 0.00604055 9.386028e-01 0.0001649487

3 roi 5 140 18.68705780 3.388674e-14 * 0.1357144798

4 group:roi 5 140 0.76745699 5.747387e-01 0.0064075258

$`Mauchly's Test for Sphericity`

Effect W p p<.05

3 roi 0.004190501 3.141115e-23 *

4 group:roi 0.004190501 3.141115e-23 *

$`Sphericity Corrections`

Effect GGe p[GG] p[GG]<.05 HFe p[HF] p[HF]<.05

3 roi 0.3648201 1.634052e-06 * 0.388899 8.291159e-07 *

4 group:roi 0.3648201 4.584366e-01 0.388899 4.657815e-01

> bl %>%

+ filter(band == "(16, 24)", time == "pre", task == "tennis") %>%

+ ezANOVA(dv=bl, wid=id, within=.(roi), between=.(group))

$ANOVA

Effect DFn DFd F p p<.05 ges

2 group 1 28 0.00149414 9.694405e-01 4.015877e-05

3 roi 5 140 18.98824468 2.190538e-14 * 1.436700e-01

4 group:roi 5 140 0.77244364 5.711403e-01 6.778804e-03

$`Mauchly's Test for Sphericity`

Effect W p p<.05

3 roi 0.002688406 1.621452e-25 *

4 group:roi 0.002688406 1.621452e-25 *

$`Sphericity Corrections`

Effect GGe p[GG] p[GG]<.05 HFe p[HF] p[HF]<.05

3 roi 0.3478699 2.251461e-06 * 0.3689256 1.231907e-06 *

4 group:roi 0.3478699 4.508580e-01 0.3689256 4.575518e-01

> bl %>%

+ filter(band == "(16, 24)", time == "post", task == "hand") %>%

+ ezANOVA(dv=bl, wid=id, within=.(roi), between=.(group))

$ANOVA

Effect DFn DFd F p p<.05 ges

2 group 1 28 0.4199406 5.222456e-01 0.011346271

3 roi 5 140 16.1221702 1.550954e-12 * 0.119091495

4 group:roi 5 140 1.0999500 3.632550e-01 0.009139281

$`Mauchly's Test for Sphericity`

Effect W p p<.05

3 roi 0.003716864 7.595502e-24 *

4 group:roi 0.003716864 7.595502e-24 *

$`Sphericity Corrections`

Effect GGe p[GG] p[GG]<.05 HFe p[HF] p[HF]<.05

3 roi 0.3547881 8.905899e-06 * 0.3770622 5.166389e-06 *

4 group:roi 0.3547881 3.347702e-01 0.3770622 3.374728e-01

> bl %>%

+ filter(band == "(16, 24)", time == "post", task == "tennis") %>%

+ ezANOVA(dv=bl, wid=id, within=.(roi), between=.(group))

Warning: Converting "id" to factor for ANOVA.

$ANOVA

Effect DFn DFd F p p<.05 ges

2 group 1 28 0.2392855 6.285328e-01 0.006820912

3 roi 5 140 17.4390996 2.124723e-13 * 0.108975540

4 group:roi 5 140 0.9038556 4.805257e-01 0.006298979

$`Mauchly's Test for Sphericity`

Effect W p p<.05

3 roi 0.003799522 9.856048e-24 *

4 group:roi 0.003799522 9.856048e-24 *

$`Sphericity Corrections`

Effect GGe p[GG] p[GG]<.05 HFe p[HF] p[HF]<.05

3 roi 0.3753116 2.483093e-06 * 0.4013268 1.250888e-06 *

4 group:roi 0.3753116 4.055686e-01 0.4013268 4.111026e-01
